# A Liquid Biopsy-Based Approach for Monitoring Treatment Response in Post-Operative Colorectal Cancer Patients

**DOI:** 10.3390/ijms23073774

**Published:** 2022-03-29

**Authors:** Barbara Kinga Barták, Tamás Fodor, Alexandra Kalmár, Zsófia Brigitta Nagy, Sára Zsigrai, Krisztina Andrea Szigeti, Gábor Valcz, Péter Igaz, Magdolna Dank, István Takács, Béla Molnár

**Affiliations:** 1 Department of Internal Medicine and Oncology, Semmelweis University, 1083 Budapest, Hungary; fodor.tamas@med.semmelweis-univ.hu (T.F.); kalmar.alexandra@med.semmelweis-univ.hu (A.K.); nagy.zsofia@med.semmelweis-univ.hu (Z.B.N.); zsigrai.sara@med.semmelweis-univ.hu (S.Z.); szigeti.krisztina_andrea@med.semmelweis-univ.hu (K.A.S.); valcz.gabor@med.semmelweis-univ.hu (G.V.); igaz.peter@med.semmelweis-univ.hu (P.I.); dank.magdolna@med.semmelweis-univ.hu (M.D.); takacs.istvan@med.semmelweis-univ.hu (I.T.); molnar.bela1@med.semmelweis-univ.hu (B.M.); 2 Molecular Medicine Research Group, Eötvös Loránd Research Network, 1083 Budapest, Hungary; 3 Department of Endocrinology, Semmelweis University, 1083 Budapest, Hungary

**Keywords:** colorectal cancer, cell-free DNA, DNA methylation, homocysteine, therapeutic response

## Abstract

Monitoring the therapeutic response of colorectal cancer (CRC) patients is crucial to determine treatment strategies; therefore, we constructed a liquid biopsy-based approach for tracking tumor dynamics in non-metastatic (nmCRC) and metastatic (mCRC) patients (*n* = 55). Serial blood collections were performed during chemotherapy for measuring the amount and the global methylation pattern of cell-free DNA (cfDNA), the promoter methylation of *SFRP2* and *SDC2* genes, and the plasma homocysteine level. The average cfDNA amount was higher (*p* < 0.05) in nmCRC patients with recurrent cancer (30.4 ± 17.6 ng) and mCRC patients with progressive disease (PD) (44.3 ± 34.5 ng) compared to individuals with remission (13.2 ± 10.0 ng) or stable disease (12.5 ± 3.4 ng). More than 10% elevation of cfDNA from first to last sample collection was detected in all recurrent cases and 92% of PD patients, while a decrease was observed in most patients with remission. Global methylation level changes indicated a decline (75.5 ± 3.4% vs. 68.2 ± 8.4%), while the promoter methylation of *SFRP2* and *SDC2* and homocysteine level (10.9 ± 3.4 µmol/L vs. 13.7 ± 4.3 µmol/L) presented an increase in PD patients. In contrast, we found exact opposite changes in remission cases. Our study offers a more precise blood-based approach to monitor the treatment response to different chemotherapies than the currently used markers.

## 1. Introduction

Cancer is one of the leading health problems with 19.3 million new cases and 10.0 million deaths worldwide in 2020 [1]. Colorectal cancer (CRC) belongs to the most common cancer types; it ranks third in men and second in women causing more than 240,000 deaths a year in Europe [2]. The key for CRC treatment is early detection, as the 5 year survival rate at stages I and II is above 60%, but after the development of distant metastases, it decreases to approximately 10% [3]. The primary goal of CRC therapy is to remove the tumor tissues with surgical intervention coupled with radio- or chemotherapy [4]. The standard chemotherapeutic agent is antimetabolite 5-fluorouracil, which can be used in combination with different agents, e.g., with oxaliplatin (FOLFOX) or irinotecan (FOLFIRI) [5]. Additionally, targeted agents can also be applied in metastatic cases, which inhibit cell proliferation, differentiation, or migration [5]. The monitoring of the therapeutic response with imaging tools (e.g., MRI, CT, or PET/CT) is a critical step in the management of treatment strategies, applying a standardized evaluation according to the response evaluation criteria in solid tumors (RECIST) [6]. Furthermore, the measurement of serum tumor markers, such as carcinoembryonic antigen (CEA) or cancer antigen 19-9 (CA 19-9), is also essential [7]. Although their sensitivity is limited separately, their simultaneous application is favorable in treatment monitoring and recurrence detection [8,9]. In the past few years, blood-based liquid biopsies—especially the cell-free DNA (cfDNA)—have received widespread attention as they can be used for cancer detection, to monitor the efficacy of therapies, or to predict metastasis formation [10]. Several studies have revealed the quantitative changes of cfDNA during the CRC development [11,12,13], and the fluctuation of its level along the treatment is also a hot research topic [14,15]. The examination of cfDNA provides an opportunity to analyze the driver mutations, including *KRAS*, *BRAF*, *APC*, or *TP53*, that can occur in the tumor tissue [16,17]. The cfDNA-based diagnostic sensitivity is relatively low in the early stages of CRC, but increases in late stages [18,19]. The analysis of the *KRAS* gene is essential to select the proper therapy, as patients harboring a *KRAS* mutation show resistance to the anti-EGFR antibody treatments [20]. The *KRAS* mutation can be used as a prognostic marker [21], for therapy monitoring [22], and also as a strong predictor of CRC recurrence [21,23]. However, due to its limited presence in the CRC population (30–40%), it is necessary to identify additional sensitive CRC-specific markers [14,16,23]. DNA methylation alterations also have a central role in cancer formation and can be investigated in cfDNA fraction. These tumor-related changes are characterized by global DNA hypomethylation at the whole genome level and promoter-specific DNA hypermethylation of certain genes [24]. An accepted approach for estimating global DNA methylation level is to determine the methylation pattern of the long-interspersed nuclear element 1 (LINE-1) retrotransposons, as approximately 17–25% of all methylation sites are located in these sequences [25,26]. In addition to the LINE-1 hypomethylation being associated with increased CRC risk, it can also be a useful prognostic marker [27]. One component of the DNA methylation process is homocysteine (HCY), which is generated from methionine and transformed into S-adenosylmethionine (SAM). SAM—the primary methyl donor molecule—is converted to S-adenosylhomocysteine (SAH) after the methyl group transfer to the cytosine–guanine (CpG) sites of DNA [28]. The elevated HCY level leads to decreased SAM/SAH ratio, which determines the methylation potential [29,30,31,32]. Therefore, the above-mentioned observations have encouraged us to examine the global DNA methylation and HCY levels simultaneously. DNA hypermethylation in the CpG islands of promoters can affect tumor suppressor genes, thereby causing reduced or silenced mRNA expression [33,34]. Several markers showing elevated methylation have already been described in CRC plasma samples [35,36,37]. According to our previously published study, four (*SFRP1*, *SFRP2*, *SDC2*, and *PRIMA1*) promising markers showed increased methylation levels in the plasma of cancer patients compared to healthy controls [38]; however, the correlation of therapeutic response to the level of their DNA hypermethylation remains unknown.

In the present study, we aim to perform a longitudinal assessment of the total amount, global and local DNA methylation pattern, and the *KRAS* mutation status of cfDNA fraction in plasma samples. Moreover, we investigate the concentration of HCY, CEA, and CA 19-9 tumor markers to monitor the therapeutic response of CRC patients over diverse treatment protocols. Our principal purpose is to examine how these parameters are influenced depending on the different outcomes of the disease.

## 2. Results

### 2.1. Patient Characteristics

Altogether, 55 cancerous patients were involved in our study, and 367 plasma samples were analyzed (Supplementary Table S1). Thirty-two patients were characterized with non-metastatic CRC (nmCRC) receiving adjuvant chemotherapy; of these people, twenty-seven remained in complete remission (REM) at the end of the treatment, while five exhibited tumor recurrence (REC). Metastatic CRC (mCRC) was identified in 23 patients, of whom 4 patients achieved remission and remained in this status, 6 patients were classified as stable disease (SD), and 13 individuals showed progressive disease (PD) at the study end (Supplementary Figure S1).

### 2.2. Alterations in Cell-Free DNA Level

**Figure 1:**
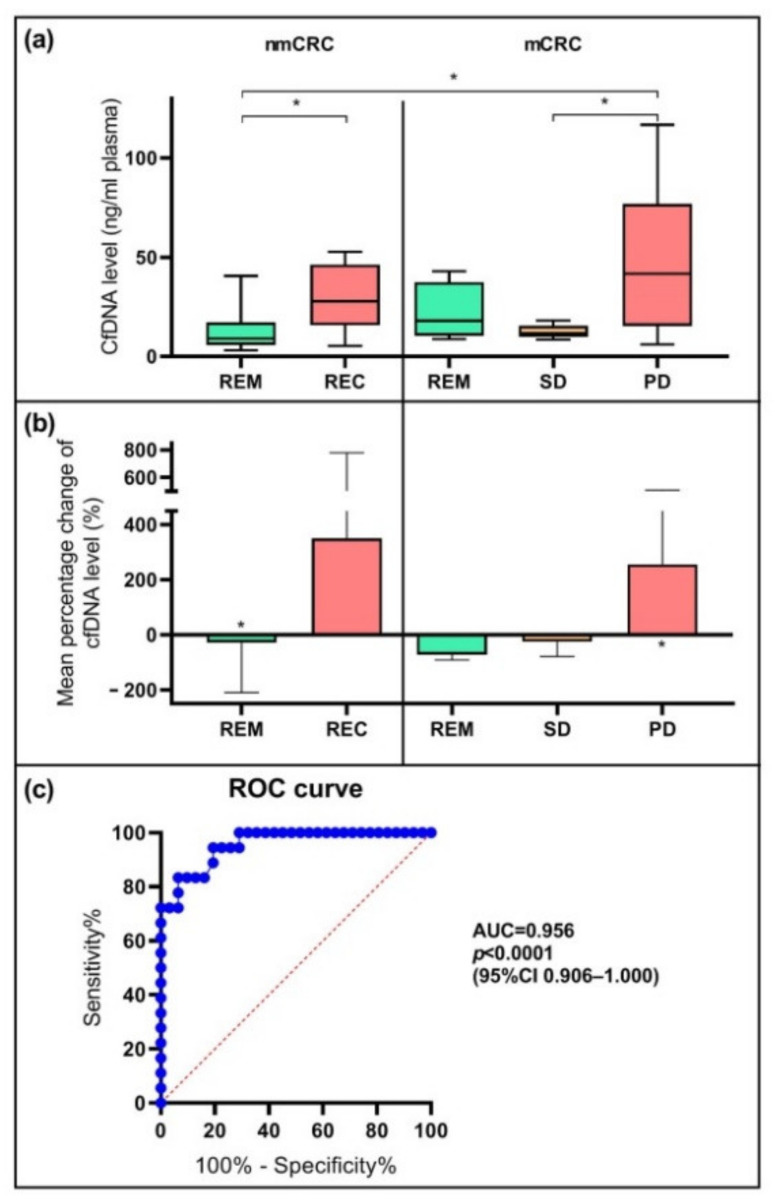
The diagnostic power of cfDNA. (**a**) Mean cfDNA amount (ng/mL plasma) in the plasma of CRC patient subgroups. Significantly different (* *p* < 0.05) cfDNA levels were noticed comparing recurrence (REC) and progressive disease (PD) vs. remission (REM) cases and progressive disease vs. stable disease (SD) among patients with metastatic CRC. (**b**) The mean percentage change (%) of cfDNA from first to last sample collection in the different CRC subgroups. Significant (* *p* < 0.05) cfDNA level elevation was observed in the case of PD between the first and last sample collection time, while an opposite trend was described in individuals with REM. (**c**) ROC curve analysis of cfDNA amount measured at study end indicated a sensitivity of 83% and specificity of 94% (AUC = 0.956, 95%CI 0.906–1.000, *p* < 0.0001) for the discrimination between individuals achieving remission and patients showing tumor progression.

The average cfDNA amount in nmCRC patients was 13.2 ± 10.0 ng in the case of remission, while in patients with REC, we detected significantly (*p* < 0.05) higher level (30.4 ± 17.6 ng). Regarding the metastatic group, the mean cfDNA quantities were 21.9 ± 14.8 ng, 12.5 ± 3.4 ng, and 44.3 ± 34.5 ng in 1 mL plasma of the patients showing remission, SD, and PD, respectively (Figure 1a). The average cfDNA levels measured at the study beginning and the end are detailed in Table 1. During the examination of the predictive power of cfDNA, we defined the degree of changes between the values determined at the start and the end of our study. A >10% alteration between the baseline and end-line was considered as “decreasing” or “increasing”; and “no change” meant <10% change in the cfDNA levels [39]. A reduced cfDNA concentration was observed in 67% of the nmCRC patients with REM, while in the case of recurrence, cfDNA quantity increased in all cases. In the mCRC patient set, we noticed a reduction in cfDNA concentration in all individuals with REM and all but one patient with SD; in contrast, 92% of PD cases presented elevation of the cfDNA level. The relative percentage change of cfDNA quantity in the patient groups between the baseline and study end is represented in Figure 1b. In remission and SD, the percentage changes of the mean cfDNA level were −27.1% * in nmCRC REM, −71.1% in mCRC REM, and −25.0% in SD. However, in cancer patients with recurrence and PD, the mean percentage variations were +350.2% in the case of REC and +255.8% * in PD (* *p* < 0.05). Receiver operating characteristic (ROC) curve analysis was performed using the values measured at the study end. We detected an effective differentiation between patients achieving remission and showing tumor progression, using the cfDNA amount of 16 ng/mL plasma as a cut-off with 83% sensitivity and 94% specificity (*p* < 0.0001) (95%CI 0.906–1.000, AUC = 0.956) (Figure 1c).

### 2.3. Changes in Genome-Wide DNA Methylation and Homocysteine Level

**Figure 2:**
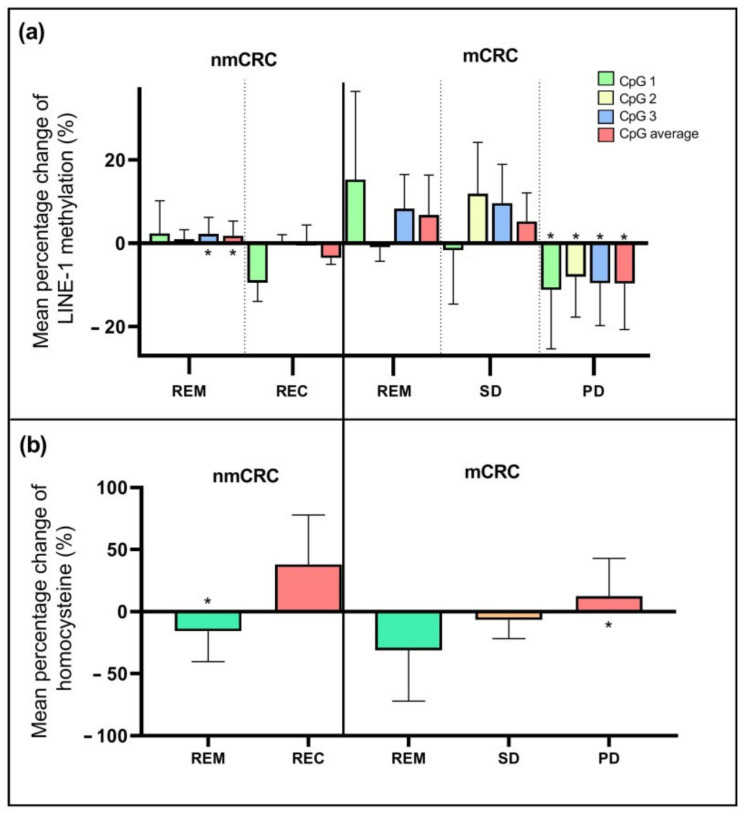
Examination of LINE-1 methylation and homocysteine level in plasma samples. (**a**) Mean percentage change (%) of LINE-1 methylation per CpG positions separately and their average from the first to the last sample collection in CRC patients with different treatment responses. In the case of progressive disease, the hypomethylation of both separate CpG sites and their average was observed, while in nmCRC patients with remission, increased DNA methylation was recognized (* *p* < 0.05). (**b**) Mean percentage change of homocysteine amount (%) from the beginning to the end of our study in CRC subgroups. The homocysteine level of the PD patients showed an elevation; in contrast, in the case of remission, a reduction was identified (* *p* < 0.05).

The cfDNA samples of 28 patients were available for global methylation measurements. The average LINE-1 methylation presented a significantly lower level (*p* < 0.05) in the case of PD (71.0 ± 6.7%) compared to nmCRC patients with REM (78.9 ± 2.0%). The methylation values at the first and last sample collection time are reported in Table 1. The relative changes of methylation are illustrated by the three examined CpG sites of LINE-1 separately, and the mean methylation level is also indicated (Figure 2a). Regarding remission cases in nmCRC, significant methylation elevations (*p* < 0.05) of the CpG3 position (+3.7%) and also the average of CpG sites (+3.0%) were observed between the beginning and the end of the study. In contrast, in the PD group, a significant decrease (*p* < 0.05) in methylation was detected in all CpG positions (mean percentage change: CpG1: −11.2%, CpG2: −8.1%, CpG3: −9.6% and CpG average: −9.7%).

The HCY concentration was determined in samples of all CRC patients. The average HCY level showed no significant differences in non-metastatic or metastatic cancer cases compared to the sample groups. However, the mean relative change between the baseline and study end (Figure 2b) revealed a significant (*p* < 0.05) increase (+12.5%) in the case of PD and a decrease in nmCRC patients with REM (−15.8%). Interestingly, these alterations showed an opposite trend with the global DNA methylation, suggesting a linkage between these parameters. The HCY levels measured at the baseline and the study are indicated in Table 1.

### 2.4. Correlation between cfDNA, LINE-1, and Homocysteine Levels

**Figure 3:**
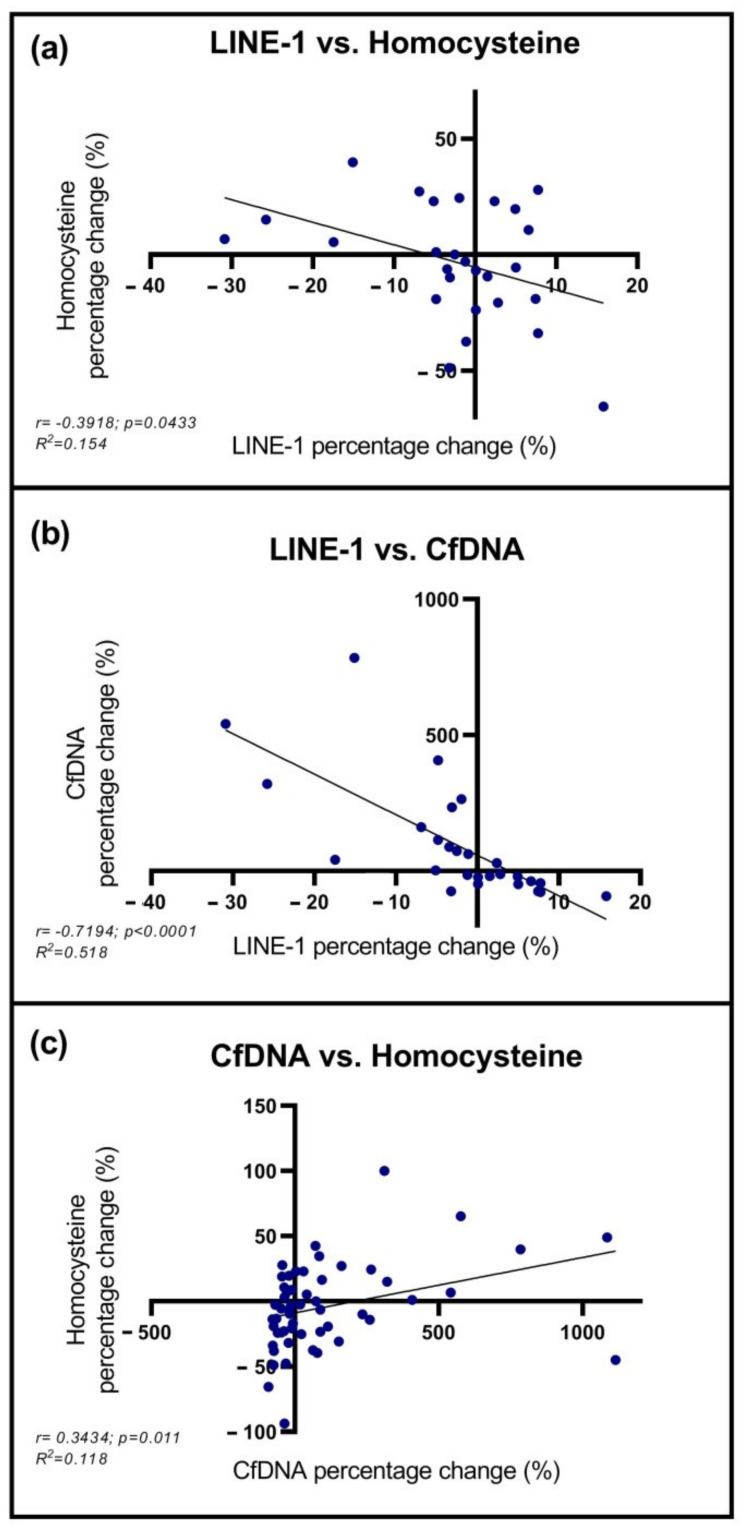
Correlation analyses of LINE-1 methylation status, homocysteine, and cfDNA levels. (**a**,**b**) A negative correlation was detected between the percentage change of mean LINE-1 methylation vs. homocysteine and cfDNA level. (**c**) A positive correlation was noticed comparing cfDNA and homocysteine percentage change.

Correlation analyses were performed between the parameters mentioned above. Mean LINE-1 methylation percentage change inversely correlated with homocysteine level changes (r = −0.3918; *p* = 0.0433), and also with the alterations of cfDNA amount (r = −0.7194 *p* < 0.0001) (Figure 3a,b). Furthermore, a positive correlation was noticed between cfDNA and HCY percentage change (r = 0.3434; *p* = 0.011) (Figure 3c).

### 2.5. DNA Methylation Pattern of SFRP2 and SDC2 Genes

**Figure 4:**
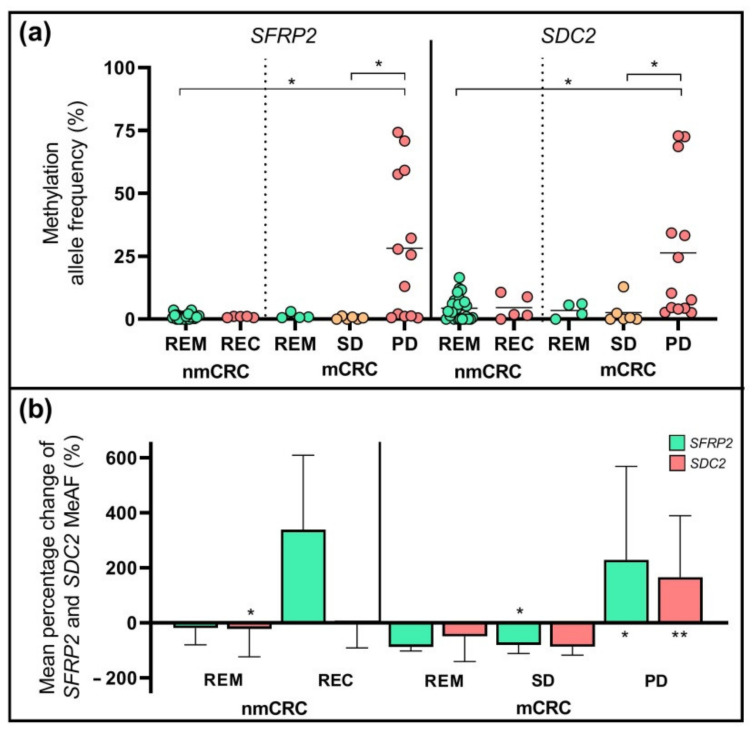
Methylation allele frequencies (MeAF) of *SFRP2* and *SDC2* genes. (**a**) The MeAF of *SFRP2* and *SDC2* at the time of last samples collection (* *p* < 0.05). (**b**) The mean percentage changes of MeAF between the beginning and end of the study. The *SFRP2* MeAF was significantly decreased in the case of stable disease, while increased in PD. In the case of *SDC2*, a reduction was observed in the nmCRC REM subgroup and a rise in the PD set (* *p* < 0.05, ** *p* < 0.005).

The methylated allele frequency (MeAF) of two selected markers, the *SFRP2* and *SDC2* were determined with droplet digital PCR (ddPCR) in plasma specimens. Methylated *SFRP2* and *SDC2* copies were detected in 87% (48/55) and 89% (49/55) of samples collected for the first time after the surgery. The MeAFs were not significantly different in subgroups at the start of the chemotherapy; however, at the study end, the amount of both methylated *SFRP2* and *SDC2* copies were significantly higher (*p* < 0.05) in the case of PD compared to the SD and REM subgroups (Figure 4a). The mean relative alterations of the *SFRP2* and *SDC2* MeAFs between the first and the last sample collection time points are illustrated in Figure 4b. The mean percentage variations of methylated *SFRP2* allele frequencies were elevated in the REC (+338.4%) and PD (+228.8% *) groups, while decreased in the nmCRC REM (−19.5%), mCRC REM (−87.9%), and SD (−79.8% *) categories (* *p* < 0.05). In the case of *SDC2*, we detected a MeAF reduction in all patient sets, except in the PD group, where a significant increase was noticed at the study end compared to the baseline (+166.1%) (** *p* < 0.005).

### 2.6. Multivariable Analysis of Selected Parameters

To predict the probability of disease outcome, multivariable logistic regression analysis was performed. Four selected parameters were involved in this analysis: the mean percentage changes of cfDNA, homocysteine, *SFRP2* MeAF, and *SDC2* MeAF between the baseline and study end. The McFadden pseudo R2 was 0.540 and indicated a good fit of the statistical model. Supplementary Table S2 contains the odds ratios (OR), 95% confidence intervals, and *p*-values, which indicate the parameters’ reliability. According to the ROC curve analyses, the sensitivity and specificity of logistic regression were 94.1% and 74.1% at the threshold determined by the Youden index with 0.781 cut-off value and 0.924 area under the curve (AUC) (Supplementary Figure S2). These values for the parameters are separately shown in Supplementary Table S2.

### 2.7. Analysis of CEA and CA 19-9 Tumor Markers

The CEA and CA 19-9 markers were measured in serum samples. In the nmCRC group, CEA remained within the reference range in 25 patients with REM (93%), while among patients with recurrent cancer, only 1 person showed an increased level. In distant metastases, the CEA concentration was higher than 5 ng/mL in at least one blood sample in 75%, 67%, and 85% of patients with REM, SD, and PD, respectively. Raised levels of CA 19-9 were detected in one person of the nmCRC group who achieved remission, but CA 19-9 did not exceed the reference value for the patients with recurrence. Furthermore, CA 19-9 levels higher than 37 U/mL were found in only 62% of mCRC patients showing PD. Significant differences were not observed between the subgroups by examining the values measured at the first and last sample collection time. The increase in CEA and CA 19-9 from first to last sample collection was more than 10% in 69% and 62% of patients with PD, respectively. Concerning the mean relative changes (%) of the tumor markers, an elevation was detected in the PD group regarding the CEA (mean percentage variation: +623%, *p* < 0.05); however, CA 19-9 did not show significant alterations in either group.

### 2.8. Association between the Analyzed Parameters and the Clinicopathological and Demographic Factors of CRC Patients

The levels of cfDNA and HCY, the methylation allele frequencies of SFRP2 and SDC2, and the CEA and CA 19-9 quantities were examined based on the clinicopathological and demographic characteristics (Table 2). The parameters measured upon first and last sample collection time and the averages of all values quantified during the study were compared. No significant differences were found based on age and gender, except for the HCY amount, as we observed an increased level in men compared to women. Higher plasma HCY was also detected in colon-located tumors compared to rectum tumors. Furthermore, almost all parameters—except for HCY—showed a significantly (*p* < 0.05) elevated level in patients with distant metastasis than cases without metastasis.

### 2.9. KRAS Mutation Analysis

**Figure 5:**
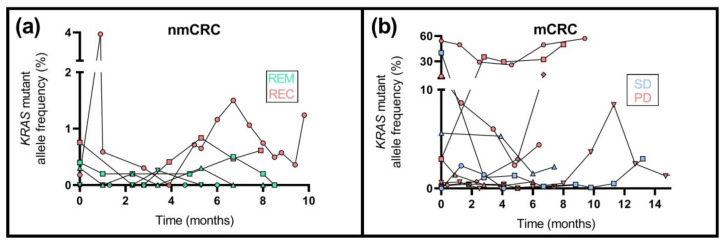
The longitudinal assessment of mutant allele frequency of the *KRAS* gene in non-metastatic and metastatic CRC patients. (**a**) In the nmCRC subgroup, the MAF was lower than 0.5% in all plasma specimens of patients with tumor remission; in contrast, a moderately higher MAF was detected in the case of recurrence. (**b**) In the case of metastatic CRC, all the individuals achieving remission had wild-type *KRAS*. Patients with progressive disease showed significantly (*p* < 0.05) higher average MAF than patients with stable disease.

*KRAS* mutation detection was performed previously on the formalin-fixed paraffin-embedded tissues of 36 from the total of 55 patients. Fifteen samples (nmCRC: *n* = 7; mCRC: *n* = 8) were found to be wild type, while *KRAS* mutations were observed in twenty-one (nmCRC: *n* = 8; mCRC: *n* = 13) specimens (Supplementary Table S1). Plasma samples of patients with both wild-type and mutated KRAS gene were examined with the ddPCR method. Mutations were not detected in plasma specimens of patients possessing wild-type tumors at all. Eighteen patients were characterized with one of the analyzed seven different *KRAS* mutations providing the Bio-Rad KRAS G12/G13 Screening Kit. Except for one patient, enough cfDNA amounts were available for the longitudinal analysis of mutation detection (Figure 5, Supplementary Table S1). The mutant allele frequency (MAF) was below 0.5% in all plasma samples of individuals without metastasis obtaining remission after the surgery (Figure 5a), while in patients with tumor recurrence, MAF was moderately elevated (mean ± SD: 0.8 ± 0.2%). Regarding the mCRC group (Figure 5b), all patients who achieved remission were characterized with wild-type *KRAS*. In the case of SD, four from six patients carried mutant *KRAS* with 3.6 ± 1.8% average MAF. Furthermore, in CRC patients showing disease progression with the mutated *KRAS* gene (9/13 patients), significantly higher (*p* < 0.05) average MAF was noticed (mean ± SD: 11.1 ± 17.4%) compared to the SD group.

## 3. Discussion

Over the past decades, several studies demonstrated that cfDNA analysis is a promising tool for predicting, detecting, and monitoring CRC. In addition to the elevation of its level, the alterations of its mutation and methylation profiles in cancer also make it an ideal biomarker [12,13,40]. Monitoring the treatment response during chemotherapy is crucial in terms of disease outcome. The currently used RECIST evaluation is based on imaging techniques with several limitations [6,41]. For these reasons, in the present study, we examined and compared the cfDNA amount, its global and local methylation pattern, and the *KRAS* mutation status in the plasma of non-metastatic and metastatic CRC patients. Furthermore, we analyzed the homocysteine level and CEA and CA 19-9 markers to monitor the disease course during the chemotherapy treatment.

According to our results, the concentration of cfDNA changed considerably during chemotherapy. We noticed >10% elevation in 92% of PD set and in all nmCRC patients showing recurrence, while a decline was detected in most individuals with remission (67% of nmCRC and 100% of mCRC). In contrast, the CEA and CA 19-9 tumor markers showed increased levels in merely 69% and 62% of patients with PD and 80% of patients with recurrent cancer. A study analyzing several tumor marker levels, including CEA, demonstrated that, after three cycles of XELOX combination chemotherapy, the CEA values did not elevate significantly in the PD group [42]. Additionally, they found increased CA 19-9 level in both partial remission and PD cases. Interestingly, our observations also indicated elevated CEA and CA19-9 in 67% and 33% of nmCRC patients achieving remission, respectively. Similar to our results, Berger et al. observed a significantly elevated cfDNA concentration in the case of disease progression compared to the “upon treatment” time point (3.6 ± 0.15 weeks after treatment initiation) in both first and second line chemotherapy treatment [39]. Moreover, at the time of disease progression in the first line treatment, 92% of cases showed a ≥10% increase in cfDNA levels, compared to CEA, where this elevation could be detected in 83% of the cases. Accordingly, cfDNA seems to outperform the studied tumor markers in reflecting the disease course, considering the direction of the change in cfDNA amount along with the therapy. However, it is necessary to note that the cfDNA concentration varies considerably across the individuals in a wide range (from 1–2 ng/mL to more than 200 ng/mL). In healthy controls, the cfDNA concentration is substantially lower than in cancer patients [16,38,43,44], and even after the development of cancer, its level is influenced by several further factors. CfDNA amount correlates with the tumor mass and stage, and it depends on the degree of the tumorous tissue vascularity and necrosis [45,46]. Additionally, additional parameters, such as age, sex, body mass index, or physical activity, also modify the amount of cfDNA [47,48]. Therefore, it is preferable to monitor the alteration’s intensity and orientation and not only focus on the absolute amount of cfDNA.

It is well known that the LINE-1 sequences in cfDNA fraction have a more pronounced hypomethylation in CRC compared to healthy controls [49]. In addition, Sunami et al. observed continuously decreasing global DNA methylation levels during CRC progression [50], and further studies reported associations between LINE-1 hypomethylation and tumor invasiveness and poor prognosis [27,51,52]. These results inspired us to examine the LINE-1 methylation changes in patients with different treatment responses. Our measurements indicated a significantly lower average global methylation in PD patients compared to individuals with REM. Furthermore, the alterations of methylation levels were noticed between the beginning and the end of the study depending on the therapeutic response status, as we observed an elevation of DNA methylation in the REM and SD groups, while we found a significant decline in its level in the PD set (from 75.5 ± 3.4% to 68.2 ± 8.4%). DNA methylation is regulated by the methionine cycle that provides SAM as the principal methyl donor molecule that is formed from methionine and adenosine triphosphate. After the donation of the methyl group by DNA methyltransferases (DNMT), SAM is converted into SAH, which then hydrolyses to homocysteine [29]. Finally, homocysteine can be recycled into methionine or converted into cysteine. Elevated HCY levels were observed in various diseases compared to healthy controls, including psoriasis, type 2 diabetes mellitus, and several age-related disorders, such as cardiovascular diseases or different cancers [53,54,55,56]. According to our findings, nmCRC patients showing remission had a significantly decreased plasma HCY level at the study end compared to baseline (−15.8%). In comparison, a significant increase (+12.5%) was noticed in the case of PD patients (*p* < 0.05). These alterations were inversely correlated with the global cfDNA methylation level changes, which means elevated HCY level can be coupled with genome-wide cfDNA hypomethylation. This association may be explained by the fact that the SAH amount is also elevated due to the high HCY concentration, leading to decreased SAM/SAH ratio. SAH molecules are strong inhibitors of DNMTs, because SAM and SAH have nearly the same chemical structures, and thus SAH can bind to the active site of the enzymes. As a consequence, the methylation potential is reduced, namely DNA hypomethylation can be observed [57]. This hypothesis is consistent with the observations of Yi et al., as they found a strong negative correlation between total HCY level and SAM/SAH ratio [31]. Furthermore, another study showed that DNA hypomethylation in the colonic mucosa positively correlated with HCY in colorectal adenoma and cancer patients [58]. An *in vitro* analysis also demonstrated genome-wide DNA hypomethylation in vascular smooth muscle cells cultured with a high level of HCY [59]. In addition to the several observations mentioned above describing the linkage between elevated HCY level and global DNA hypomethylation, according to our best knowledge, this is the first study that has focused on the methylation level alterations of cfDNA in connection with the HCY amount to monitor therapeutic response in CRC. Based on our results, elevated HCY in patients with PD is also accompanied by increased cfDNA level. These results are consistent with Li et al., as they observed significantly higher cfDNA levels in patients with essential hypertension and hyperhomocysteinemia than individuals without elevated HCY [60]. Moreover, they have revealed that neutrophil granulocytes in patients with high HCY level can enhance a process called NETosis. During this process, neutrophil extracellular traps (NETs) consisting of disintegrated chromatin are released from neutrophils to trap and kill microorganisms; furthermore, they are also involved in thrombosis formation [60]. NETosis is considered an active DNA secretion method that can contribute to high cfDNA level in addition to different passive mechanisms [61]. The above-mentioned observations may indicate that, during cancer progression, elevated HCY level can promote cfDNA increase through NETosis; moreover, it may also influence global DNA methylation pattern through the methionine cycle. In addition to global DNA hypomethylation, promoter-specific DNA hypermethylation is also characteristic in CRC. We have previously demonstrated the significantly elevated methylation levels of four genes, including *SFRP2* and *SDC2*, in the case of CRC compared to healthy controls [38]. We observed methylated *SFRP2* and *SDC2* in 72.3% and 89.4% of the CRC patients. The methylation levels of these markers are affected by the differentiation status, the TNM stage, and the number of lymph node metastasis [62,63]. Nevertheless, the dynamics of these markers’ alterations depending on the disease outcome are still unknown. In the present study, we detected methylated copies of both genes at the first sample collection time in more than 85% of the patients. At the last sample collection time, the MeAFs of both markers were significantly higher in patients with PD in comparison with remission cases (*p* < 0.05); however, it showed a moderate variability across the patients. This fact requires further studies to evaluate the underlying biological phenomenon. The mean percentage change of MeAFs was significantly elevated in the PD group in both *SFRP2* and *SDC2* and has shown a decrease in REM and SD patients. These observations suggest that the methylation pattern of these genes did not change shortly after surgery, but the alteration of MeAF along chemotherapy may reflect the different disease outcomes. Barault et al. identified five CRC-specific methylation markers, and they found that the samples collected close to documented tumor progression time revealed a non-significant increase in the methylation level of all markers [14]. Moreover, in patients receiving conventional chemotherapy, the average of selected markers indicated the tumor burden changes, as it decreased in partial remission or SD, while increased in PD.

We performed multivariable logistic regression analysis to predict the efficacy of the combined use of cfDNA amount, HCY level, *SFRP2*, and *SDC2* MeAF alterations during the chemotherapy. ROC curve analyses showed the highest AUC (0.924) for the logistic regression, while lower values (0.887; 0.815; 0.776; 0.808) were calculated for the separate parameters, respectively. The sensitivity (94.1%) and specificity (74.1%) of the optimal point for the multivariable analysis show that the percentage changes of the involved parameters may indicate the treatment response. By analyzing the variables individually, the alteration of cfDNA level was able to approximate the efficiency of our combined model with 88.2% sensitivity and 85.2% specificity (AUC: 0.887). However, increasing the number of patients with different disease outcomes would be beneficial to enhance our model’s reliability.

Regarding the experiments on *KRAS* mutation status, only plasma samples of patients with determined tissue mutation status were included in our *KRAS* mutation plasma analysis (36/55). In patients with wild-type *KRAS* tumors (15/36), mutant copies in plasma samples were not observed. Among *KRAS* mutant patients, during postoperative chemotherapy, the MAF was below 0.5% in all plasma samples in patients who achieved REM, and after the final chemotherapy treatment, mutant copies were not detectable at all. On the other hand, moderately higher MAF was detected in the case of recurrence. Furthermore, we observed mutant copies with various MAFs in SD and PD patients, but there were no significant differences between the baseline and the study end. These observations are similar to the results of Klein-Scory et al., as they showed a rapid disappearance of *RAS* mutation in the plasma of CRC patients with partial remission and SD after the first cycles of chemotherapy, while in the case of progression, they did not find any decrease [64]. Another study has also shown the reduction in *RAS* mutation load in patients responding to systematic therapy following 8–12 weeks of treatment [65]. These findings suggest that the blood-based analysis of mutant copies in patients with *KRAS*-mutant tumors can be a potential tool for tracking tumor development, but further examinations are needed. Moreover, according to the limited presence of *KRAS* mutation in CRC patients (30–40%) [14,16,23], it is recommended to focus on markers that can be used more widely in this population.

Our study presented a comprehensive liquid biopsy-based analysis focusing on markers that are influenced by the different outcomes of colorectal cancer. However, this single-center study has some limitations, including the moderate sample sizes of some clinical subgroups. Additionally, the low cfDNA amount of certain samples could not allow us to perform the methylation measurements in the case of all patients. Furthermore, as multiple markers are examined, the automation of the different steps would facilitate the implementation of our analyses in clinical practice. Despite these limitations, we assume that our findings regarding the alterations of the investigated parameters in patients with different treatment responses are sufficiently substantiated, robust and reliable.

## 4. Materials and Methods

### 4.1. Patient Inclusion and Sample Collection

We enrolled 55 CRC patients in our study at the Department of Internal Medicine and Oncology, Semmelweis University in Budapest, Hungary. Clinicopathological features of the patients can be found in Supplementary Table S1. Thirty-two patients with stage II and III CRC were treated with adjuvant chemotherapy, while twenty-three individuals received treatment for metastatic disease. Post-operative blood samples (*n* = 367) were collected from each CRC patient before and during the chemotherapy. The sample collection was carried out every four weeks on average immediately before the chemotherapy treatment following overnight fasting. We excluded patients whose primary cancer was not colorectal cancer and those from whom less than five plasmas could be collected. Patients were classified based on their treatment response status determined by an expert radiologist after the last sample collection with imaging techniques (PET-CT/CT/MRI) (Supplementary Table S1). In the case of adjuvant therapy, we distinguished individuals who remained in remission and who exhibited tumor recurrence. Patients with distant metastasis were categorized into three groups: (1) achieved remission (partial or complete), (2) had stable disease, and (3) had progressive disease. The first blood sampling (termed as baseline) was immediately before the first chemotherapy treatment following the surgery. The last blood sample (termed as study end) was taken at the time of the final chemotherapy in patients with remission, while in other cases, the study lasted for an average of ten months depending on the availability of samples, or until the patient’s death. The study was approved by the local ethics committee and government authorities (Regional and Institutional Committee of Science and Research Ethics; TUKEB Nr: 14383-2/2017/EKU). Written informed consent was obtained from all patients before sample collection. Blood samples were processed within 4 h of collection by double centrifugation at 1350 rcf for 12 min, and plasma fractions were stored at −80 ˚C.

### 4.2. Cell-Free DNA Isolation and Bisulfite Conversion

CfDNA was isolated from 4 mL plasma samples using Quick-cfDNA Serum & Plasma Kit (Zymo Research, Irvine, CA, USA), according to the manufacturer’s instructions, and it was eluted in 50 µL Elution Buffer. The sample concentration was measured with Qubit 1.0 fluorometer using Qubit dsDNA High Sensitivity Assay Kit (Thermo Fisher Scientific, Waltham, MA, USA). The bisulfite conversion of 50 ng cfDNA was performed using EZ DNA Methylation Direct Kit (Zymo Research) according to the manufacturer’s instructions with the elution volume of 20 µL.

### 4.3. Global DNA Methylation Level Analysis

To estimate the global DNA methylation level, the methylation pattern of three CpG-sites located in the LINE-1 sequences was determined in the plasma samples of 13 patients with nmCRC and 15 people with mCRC. In brief, 20 ng bisulfite converted DNA was used as a template to amplify the 146 bp length LINE-1 region using the PyroMark PCR Kit (Qiagen, Hilden, Germany). After preparing the PCR product, PyroMark Q24 system (Qiagen) was used for pyrosequencing according to the PyroMark Q24 CpG LINE-1 Handbook (Qiagen). The quantification of LINE-1 methylation was performed with the PyroMark Q24 Software (Qiagen). The mean LINE-1 methylation was calculated as the average of the three analyzed CpG position methylation percentages. The relative change of LINE-1 methylation per patient between the first and last sample collection was calculated according to the following formula: [100 × (last − first)/first] [66].

### 4.4. SFRP2 and SDC2 Methylation Analysis

Based on the previous results of our research group [38], two DNA methylation markers, *SFRP2* and *SDC2* genes were selected. Duplex ddPCR assays were designed for the two genes using PyroMark Assay Design 2.0 software (Qiagen). Primer pairs were tested *in silico* by BiSearch software [67]. Two hydrolysis probes were multiplexed, FAM-labelled for methylated and VIC-labelled for unmethylated sequences with MGB quencher. The two probes were specific for the same regions of the gene promoters. Each of the 22 µL ddPCR reaction mixture contained 2× ddPCR Supermix for Probes (no dUTP) (Bio-Rad, Hercules, CA, USA), the primers and probes in 900 and 250 nM final concentration (Thermo Fisher Scientific), and the bisulfite converted DNA template. For droplet generation, QX200 AutoDG (Bio-Rad) instrument was applied resulting in water-in-oil droplets. The amplification was performed with Mastercycler ep Gradient S instrument (Eppendorf, Hamburg, Germany) with the following PCR conditions: 95 °C for 10 min, 45 cycles of denaturation at 94 °C for 30 sec and annealing at 59 °C/61 °C for 1 min, 98 °C for 10 min for enzyme deactivation, and 4 °C for storing. The annealing temperature was determined as 59 °C in *SFRP2* and 61 °C in *SDC2* after optimization by gradient PCR. Non-templated control, 100% methylated, and 100% unmethylated DNA (EpiTect Methylated and Unmethylated Controls, Qiagen) as positive and negative controls were also amplified simultaneously. Finally, the 96-well plate with droplets was placed into the QX200 Droplet Reader (Bio-Rad). For data analyses, we used QuantaSoft Software (Bio-Rad) as recommended by the manufacturer. The methylated allele frequency (MeAF) (%) was calculated from the resulting copy number values as follows: [MET copy/(MET + UNMET copy) × 100].

### 4.5. KRAS Mutation Analysis

*KRAS* mutation status was determined from formalin-fixed, paraffin-embedded tissue by a pathologist as part of the treatment protocol. To monitor the alteration of the mutated *KRAS* copy number during the therapy, ddPCR analysis was performed on the plasma samples using the QX200 Droplet Digital PCR system (Bio-Rad). As negative controls, cfDNA samples of patients with wild-type *KRAS* tumors were also examined. *KRAS* G12/G13 Screening Kit (Bio-Rad) was applied that contains assays for detecting seven *KRAS* mutations (G12A, G12C, G12D, G12R, G12S, G12V, and G13D), providing precise detection of the mutated copies with a sensitivity of 0.2%. The PCR was carried out in 22 µL reaction volume containing 11 µL ddPCR Supermix for Probes (2×, no dUTP) (Bio-Rad), 1.1 µL multiplex primers/probes (FAM for the mutated and HEX for the wild-type alleles) (Bio-Rad), and 9.9 µL cfDNA template. The PCR protocol was the same as described above, except the annealing temperature was 55 °C. After reading the droplets, QuantaSoft Software (Bio-Rad) was used for data analysis, and the rate of mutated and wild-type copies was determined.

### 4.6. Tumor Marker and Homocysteine Level Detection

The serum CEA and CA 19-9 markers were measured with *in vitro* assays at the Department of Laboratory Medicine, Semmelweis University. Chemiluminescent microparticle immunoassays were performed using Architect i2000SR (Abbott Laboratories, Chicago, IL, USA). The normal reference values for CEA and CA 19–9 were set as 0–5 ng/mL and 0–37 U/mL, respectively. The amount of homocysteine was quantified in all blood samples with enzyme cycling assay using Roche Cobas C311 analyzer (Roche Diagnostics, Basel, Switzerland). The normal reference range differs between men (5.4–16.0 µmol/L) and women (4.4–13.5 µmol/L).

### 4.7. Statistical Analysis

For the normality test, the Shapiro–Wilk test was used. Statistical significances (*p* < 0.05) were assessed by the Kruskal–Wallis test, followed by Dunn’s test or one-way ANOVA followed by Tukey’s multiple comparisons test using Prism8 software (GraphPad, San Diego, CA, USA). In paired comparisons, Wilcoxon matched-pairs signed rank tests or Student’s *t*-test with a significance criterion (*p* < 0.05) were performed with Prism8 software depending on non-normal or normal data distribution, respectively (GraphPad). In the case of unpaired comparisons, Mann–Whitney test or unpaired *t*-test was applied. Prism8 software (GraphPad) was used for ROC curve analysis and determination of sensitivity and specificity. After calculating Youden’s index, the cut-off value for the highest sensitivity was selected. Multivariable logistic regression was performed to predict the effect of mean percentage changes in the selected parameters (cfDNA, homocysteine, *SFRP2*, and *SDC2* methylation) on disease outcome. Global DNA methylation was excluded from this analysis due to missing values. Additionally, as the *KRAS* mutant allele occurred in only 58% of the patients, the mutant allele frequency was also excluded from this calculation. To evaluate the goodness of fit of the statistical model, McFadden pseudo R2 was calculated, and the predictive performance of the parameters was measured by plotting ROC curves and calculating AUC.

## 5. Conclusions

The present study offers the possibility to monitor the therapeutic response during chemotherapy with a minimally invasive blood-based method applying the alterations of cfDNA amount, global and local DNA methylation pattern, and HCY level. Digital PCR technique provides a highly sensitive method for examining genetic and epigenetic patterns; however, as KRAS mutation frequency is relatively low in CRC cases, its detection has limited diagnostic applicability. On the other hand, gene-specific methylation may become a more promising, widely used marker. This study also highlighted a possible connection between the elevated homocysteine concentration, the reduced global DNA methylation level, and the increased cell-free DNA amount in CRC patients with progressive disease.

## Figures and Tables

**Table 1:** The level of cfDNA, global cfDNA methylation, and homocysteine at the first and last sample collection time. The data represent mean ± SD. Statistically significant (*p* < 0.05) differences between the values measured at the study beginning and the end are highlighted in bold. The * and ** mean significant differences (*p* < 0.05) in the comparisons of REM vs. REC in nmCRC and REM vs. PD in mCRC patients, respectively (cross-sectional comparison).

			CfDNA (ng/mL Plasma)	Mean LINE-1 Methylation (%)	Homocysteine (µmol/L)
**nmCRC**	REM	Baseline	**10.1 ± 6.3**	**78.2 ± 1.3**	**13.3 ± 3.4**
		Study end	**6.1 ± 2.8**	**80.5 ± 2.8**	**10.8 ± 3.0**
	REC	Baseline	14.9 ± 15.9	81.3 ± 3.3	10.1 ± 0.9
		Study end	32.3 ± 22.1 *	78.5 ± 3.5	14.0 ± 4.1
**mCRC**	REM	Baseline	13.0 ± 5.4	74.7 ± 7.0	12.9 ± 3.9
		Study end	6.3 ± 4.2	79.6 ± 2.0	7.9 ± 2.6
	SD	Baseline	17.5 ± 13.7	74.7 ± 6.5	11.7 ± 1.6
		Study end	10.2 ± 6.0	78.4 ± 2.2	11.0 ± 2.5
	PD	Baseline	**30.6 ± 39.8**	**75.5 ± 3.4**	**10.9 ± 3.4**
		Study end	75.6 ± 69.8 **	**68.2 ± 8.4**	**13.7 ± 4.3**

**Table 2:** Association between the parameters and CRC patients’ clinicopathological and demographic factors. The data are shown as mean ± SD. The statistically significant differences between the groups (*p* < 0.05) are bold.

**Variables**	Collection	Gender		Age		Tumor Location		Distant Metastasis	
		Male	Female	<60	>60	Colon	Rectum	No	Yes
		*n* = 35	*n* = 20	*n* = 12	*n* = 43	*n* = 39	*n* = 16	*n* = 32	*n* = 23
**CfDNA**	Baseline	21.7 ± 35.8	14.3 ± 15.4	30.0 ± 45.1	15.9 ± 24.1	21.7 ± 34.6	12.5 ± 12.4	**10.6 ± 11.1**	**30.6 ± 41.4**
**(ng/mL)**	Study end	24.2 ± 41.5	28.9 ± 49.8	37.5 ± 61.1	22.7 ± 38.7	28.2 ± 49.8	20.4 ± 27.2	**11.1 ± 12.7**	**46.5 ± 60.5**
	Mean	22.5 ± 22.9	22.8 ± 23.5	30.5 ± 25.7	20.4 ± 21.8	24.8 ± 26.0	17.3 ± 11.1	**15.8 ± 12.6**	**32.1 ± 29.2**
**Hcy**	Baseline	12.8 ± 3.0	11.6 ± 3.6	11.5 ± 3.2	12.5 ± 3.4	**12.9 ± 3.5**	**10.8 ± 2.5**	12.8 ± 3.3	11.6 ± 3.2
**(umol/L)**	Study end	**11.9 ± 3.4**	**10.0 ± 3.3**	10.4 ± 3.8	11.4 ± 3.5	11.6 ± 3.5	10.3 ± 3.6	11.3 ± 3.3	11.1 ± 3.7
	Mean	**12.0 ± 2.6**	**9.8 ± 2.3**	10.5 ± 3.1	11.6 ± 2.6	11.6 ± 2.9	10.6 ± 2.2	11.9 ± 2.5	10.6 ± 2.8
***SFRP2*** **MeAF**	Baseline	10.7 ± 22.0	2.4 ± 2.7	8.4 ± 21.6	7.5 ± 17.1	7.7 ± 16.7	7.6 ± 21.4	**1.4 ± 1.6**	**16.5 ± 25.0**
	Study end	8.0 ± 18.4	6.7 ± 17.5	10.0 ± 21.6	6.8 ± 17.0	8.3 ± 20.1	5.6 ± 10.5	**1.0 ± 0.9**	**16.3 ± 24.6**
**(%)**	Mean	7.2 ± 13.7	3.4 ± 6.0	6.0 ± 8.2	5.8 ± 12.4	6.5 ± 12.9	4.2 ± 7.6	**1.1 ± 0.7**	**12.4 ± 15.4**
***SDC2*** **MeAF**	Baseline	14.5 ± 21.0	9.0 ± 10.6	12.2 ± 23.9	12.5 ± 16.4	13.2 ± 17.3	10.6 ± 20.3	**8.7 ± 9.1**	**17.7 ± 24.5**
	Study end	11.5 ± 20.3	6.7 ± 6.9	7.1 ± 6.4	10.5 ± 18.7	10.4 ± 18.6	8.3 ± 11.7	**5.1 ± 5.4**	**16.2 ± 23.4**
**(%)**	Mean	11.4 ± 14.8	8.2 ± 7.6	6.9 ± 6.0	11.2 ± 13.9	11.4 ± 14.2	7.4 ± 7.2	**8.1 ± 7.1**	**13.2 ± 17.1**
**CEA**	Baseline	48.4 ± 158	19.8 ± 55.1	93.1 ± 235	65.1 ± 268	55.4 ± 152	109 ± 422	**1.9 ± 1.2**	**105 ± 193**
**(ng/mL)**	Study end	27.9 ± 55.3	41.9 ± 138	73.0 ± 178	22.4 ± 50.6	37.2 ± 107	23.1 ± 44.4	**2.7 ± 1.9**	**82.9 ± 135**
	Mean	40.9 ± 103	17.2 ± 31.9	46.3 ± 83.6	28.7 ± 85.9	58.5 ± 188	38.8 ± 124	**2.5 ± 1.5**	**81.8 ± 122**
**CA 19-9**	Baseline	173 ± 484	88.6 ± 306	69.0 ± 147	162.4 ± 475	150 ± 466	122 ± 322	**4.8 ± 9.1**	**357 ± 617**
**(U/mL)**	Study end	165 ± 478	83.1 ± 313	128.7 ± 404	136.5 ± 433	166 ± 488	60.7 ± 188	**7.5 ± 16.3**	**335 ± 621**
	Mean	127 ± 356	46.7 ± 109	56.7 ± 108	108.6 ± 326	109 ± 327	68.0 ± 185	**6.7 ± 11.1**	**239 ± 425**

## Data Availability

Not applicable.

## Supplementary Materials

The following supporting information can be downloaded at: https://www.mdpi.com/article/10.3390/ijms23073774/s1.
