# Supplementary material for: A Liquid Biopsy-Based Approach for Monitoring Treatment Response in Post-Operative Colorectal Cancer Patients
Source: Int J Mol Sci. 2022 Mar 29;23(7):3774. doi: 10.3390/ijms23073774 (PMC8998310; doi:10.3390/ijms23073774)
Supplement: Supplementary file 1 [file ijms-23-03774-s001.zip › Supplementary Figure S1 IJMS.pdf]

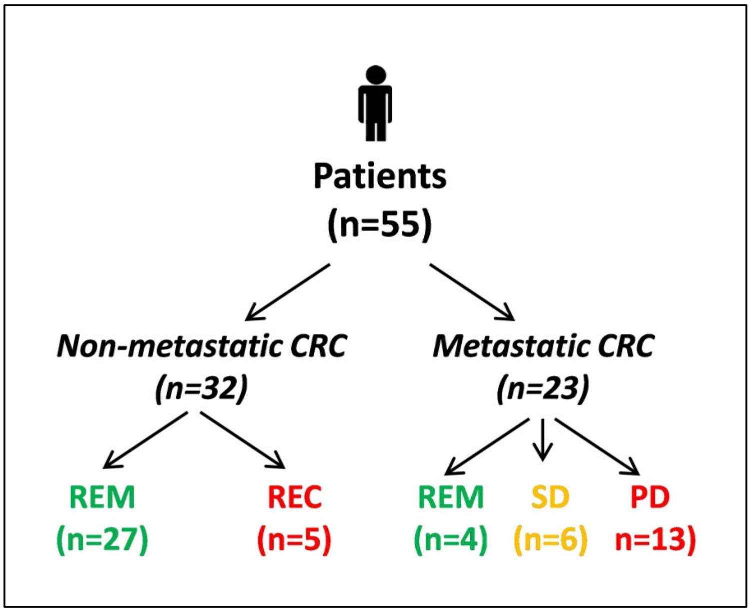

Supplementary Figure S1. Patient distribution. The CRC patients were grouped based on the disease outcome. REM: remission; REC: recurrence; SD: stable disease; PD: progressive disease.
