# Supplementary material for: A Liquid Biopsy-Based Approach for Monitoring Treatment Response in Post-Operative Colorectal Cancer Patients
Source: Int J Mol Sci. 2022 Mar 29;23(7):3774. doi: 10.3390/ijms23073774 (PMC8998310; doi:10.3390/ijms23073774)
Supplement: Supplementary file 1 [file ijms-23-03774-s001.zip › Supplementary Figure S2 IJMS.pdf]

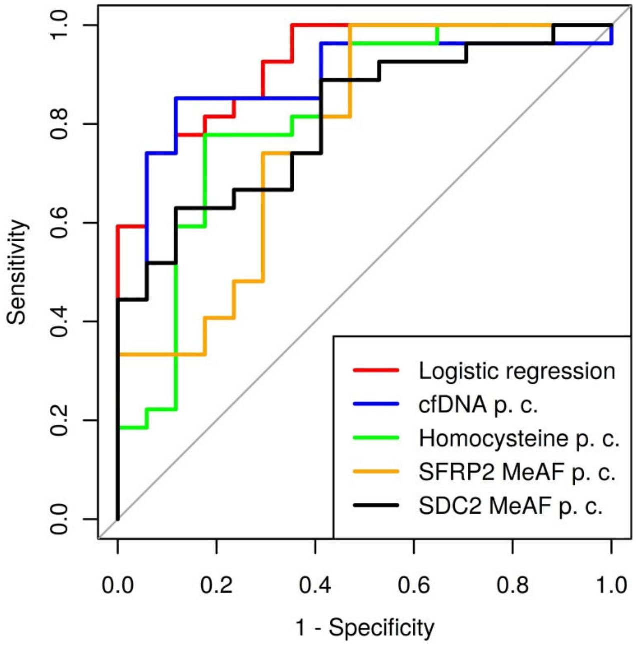

Supplementary Figure S2. ROC curve analyses of logistic regression and selected parameters separately (percentage changes of cfDNA, homocysteine, SFRP2, and SDC2 methylation). The highest AUC (0.924) was for the logistic regression followed by the other markers with 0.887, 0.815, 0.776, and 0.808, respectively. p.c.: percentage change, MeAF: methylation allele frequency.
